# Entropy Analysis and Neural Network-Based Adaptive Control of a Non-Equilibrium Four-Dimensional Chaotic System with Hidden Attractors

**DOI:** 10.3390/e21020156

**Published:** 2019-02-07

**Authors:** Hadi Jahanshahi, Maryam Shahriari-Kahkeshi, Raúl Alcaraz, Xiong Wang, Vijay P. Singh, Viet-Thanh Pham

**Affiliations:** 1 Department of Aerospace Engineering, Faculty of New Sciences and Technologies, University of Tehran, Tehran 14395-1561, Iran; 2 Faculty of Engineering, Shahrekord University, Shahrekord 64165478, Iran; 3 Research Group in Electronic, Biomedical and Telecommunication Engineering, University of Castilla-La Mancha (UCLM), 16071 Cuenca, Spain; 4 Institute for Advanced Study, Shenzhen University, Shenzhen 518060, China; 5 Department of Biological & Agricultural Engineering and Zachry Department of Civil Engineering, Texas A&M University, 2117 TAMU, College Station, TX 77843, USA; 6 Nonlinear Systems and Applications, Faculty of Electrical and Electronics Engineering, Ton Duc Thang University, Ho Chi Minh City 700000, Vietnam

**Keywords:** Non-equilibrium four-dimensional chaotic system, entropy measure, adaptive approximator-based control, neural network, uncertain dynamics

## Abstract

Today, four-dimensional chaotic systems are attracting considerable attention because of their special characteristics. This paper presents a non-equilibrium four-dimensional chaotic system with hidden attractors and investigates its dynamical behavior using a bifurcation diagram, as well as three well-known entropy measures, such as approximate entropy, sample entropy, and Fuzzy entropy. In order to stabilize the proposed chaotic system, an adaptive radial-basis function neural network (RBF-NN)–based control method is proposed to represent the model of the uncertain nonlinear dynamics of the system. The Lyapunov direct method-based stability analysis of the proposed approach guarantees that all of the closed-loop signals are semi-globally uniformly ultimately bounded. Also, adaptive learning laws are proposed to tune the weight coefficients of the RBF-NN. The proposed adaptive control approach requires neither the prior information about the uncertain dynamics nor the parameters value of the considered system. Results of simulation validate the performance of the proposed control method.

## 1. Introduction

A variety of chaotic systems with various features, such as multistability [1,2,3], extreme multistability [4,5], and multi-scroll attractors [6,7], have been introduced in recent years for investigating nonlinear dynamical systems. Dynamical systems can be categorized based on self-excited and hidden attractors [8]. From 1994, when the first non-equilibrium chaotic flow was reported in literature [9], almost 20 years have passed before another chaotic systems with non-equilibrium was introduced [10,11,12,13,14,15]. It can be easily concluded that the chaotic attractor in such systems is hidden. Given the fact that systems without equilibrium have unexpected responses to perturbations, these systems have become attractive systems for researchers.

However, all the aforementioned systems with no-equilibria are described by 3D differential equations. So, the question is if there is any 4D system with no-equilibria. The first 4D chaotic system was found by Rössler in 1979 [16], which was the first step in designing a 4D chaotic system. In the last few years, only a few works related with 4D chaotic dynamical systems with no-equilibria have been reported. In 2014, Wei et al. presented a new four-dimensional hyperchaotic system with no-equilibria developed by extension of the generalized diffusionless Lorenz equations [17]. In 2015, a no-equilibrium chaotic system with multiwing butterfly attractors constructed using a state feedback controller was proposed by Tahir et al. [18]. Motivated by complex dynamical behaviors of chaotic systems and unusual features of hidden attractors, a novel no-equilibrium chaotic system with an exponential nonlinearity was also proposed by Pham et al. in 2015 [19]. In 2016, Pham et al. introduced a novel four-dimensional continuous-time autonomous system with a cubic nonlinear term, which does not have equilibria [20]. In 2017, Bao et al. presented a memristive system, which does not display any equilibrium but can exhibit hyperchaotic, chaotic, and periodic dynamics as well as transient hyperchaos [21]. Furthermore, in 2018, Zhang et al. introduced a 4D chaotic composed of nine terms including only one constant term having also a line of equilibrium points or no equilibrium points [22].

In order to suppress the chaotic behavior of the nonlinear systems, several control methods have been implemented. Mobayen and Ma introduced a combination of finite-time robust-tracking theory and composite nonlinear feedback approach [23]. Shukla and Sharma designed a backstepping controller and analyzed the stability of the designed controller for a class of three-dimensional chaotic systems [24]. To name just a few, fuzzy controller [25,26,27,28,29], sliding mode controller [30,31,32,33,34], and hybrid controllers [35,36,37,38,39] are some other controllers that are implemented to control and synchronize the chaotic systems.

Artificial intelligence methods have been used widely to successfully solve a wide range of problems [40,41,42,43,44]. Designing the controllers based on the Neural network, as one of the most used artificial intelligence-based controllers (especially when dealing with complex nonlinear systems), is used extensively. Neural network–based control procedure can provide an efficient solution to the control of the complex, uncertain, and ill-defined systems. Some interesting results on using neural network to control and synchronize of complex systems have been studied in [45,46,47,48]. Yadmellat and Nikravesh have proposed a neural network–based output-feedback control method for nonlinear chaotic systems [49]. In another paper, Sarcheshmeh et al. designed two neural controllers to synchronize two master and slave chaotic satellites [50]. In order to suppress the disturbances in the chaotic systems, it is necessary to design an adaptive controller. In this regard, Fang et al. proposed a hybrid of an adaptive neural synchronization algorithm and a backstepping technique to synchronize a class of uncertain chaotic systems [51]. Shao et al. developed an adaptive neural network–based synchronization control strategy to stabilize a general form of unknown chaotic systems in the presence of unknown disturbances [52].

This paper focuses on the control of an uncertain four-dimensional chaotic system, which presents completely uncertain and chaotic nonlinear dynamics, such as an entropy analysis corroborates. Three well-known entropy-based metrics are computed from the time series generated by the system, thus highlighting different levels of complexity for different conditions. Since neural network is a universal approximator and it has a powerful tool for learning and approximating arbitrarily functions. Therefore, in this work, RBF-NN as a linear-in-parameter approximator has been chosen to approximate the uncertain nonlinear dynamics of the four-dimensional chaotic system. Moreover, no prior knowledge about system parameters is available. Then, the proposed indirect adaptive technique is proposed by using the developed RBF-NN-based model. Stability analysis shows that all of the closed-loop signals are semi-globally uniformly ultimately bounded and by proper choice of the design parameters the tracking error converges to the small vicinity of the origin. Also, weights of the RBF-NN are calibrated using the adaptive laws derived using the Lyapunov direct method. Simulation results verify the effectiveness of the proposed approach in control of the uncertain chaotic system with hidden attractors.

The paper is organized as follows. In Section 2, the four-dimensional chaotic system is described. In Section 3, the entropy analysis of the proposed system is presented. The RBF-NN and the design of the suggested control strategy are introduced in Section 4. In this section, the stability analysis of presented control algorithm is also discussed. The final section concludes the paper.

## 2. Four-dimensional Chaotic System

**Figure 1:**
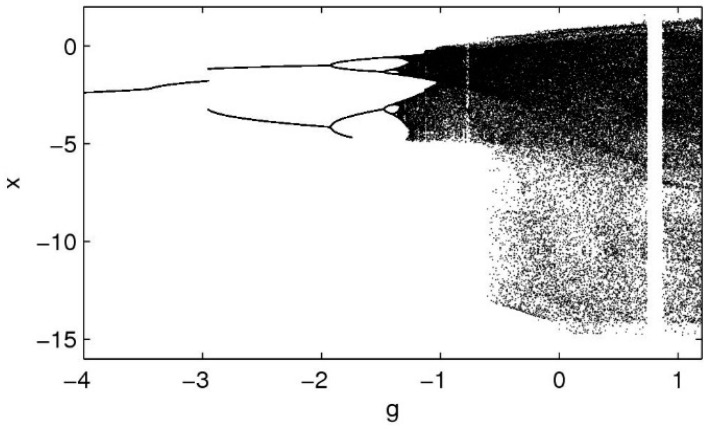
A bifurcation diagram exhibiting a periodic-doubling route to chaos of the peak of x (x max) of system (1) versus parameter g.

The general form of the proposed four-dimensional chaotic system is described as follows:

Let x, y, z, and w be the state variables of the system. Then,

(1) $$\begin{array}{c} \dot{x}=y\\ \dot{y}=z\\ \dot{z}=w\\ \dot{w}=-aw+bx^{2}-cy^{2}+exy+fxz+g \end{array}$$

where a, b, c, e, f, and g are system parameters. The behavior of the system depends on the numerical value of its parameters. The equilibrium states are found by setting the left-hand side of (1) to zero. Equation (1) gives y=z=w=0, while $bx^{2}=g$. If b and g are both nonzero with the same signs, then there are no equilibria. If g=0, then Equation (1) gives x=0, so there is the trivial equilibrium (0,0,0,0). If bg<0, there exist two equilibrium points $\left(\pm \sqrt{-g/b},0,0,0\right)$. The chaos of the dynamical system can be characterized by the Lyapunov exponent, which can be used to characterize the sensitivity of the system to the initial values. Considering Lyapunov exponents as $L_{1}$, $L_{2}$, $L_{3}$, and $L_{4}$ such that $L_{1}>L_{2}>L_{3}>L_{4}$ and assuming $L_{1}>0$, $L_{2}=0$, $L_{3}<0$, and $L_{4}<0$, the dynamical behavior of the system (1) is chaotic. Taking a=1.05, b=0.7, c=0.19, e=1.37, f=1.79, Figure 1 shows a bifurcation diagram which exhibits a periodic-doubling route to chaos of the peak of x (x max) of the system (1) versus parameter g, which is varied from −4 to 1.2. There are also some periodic windows in the chaotic region.

**Figure 2:**
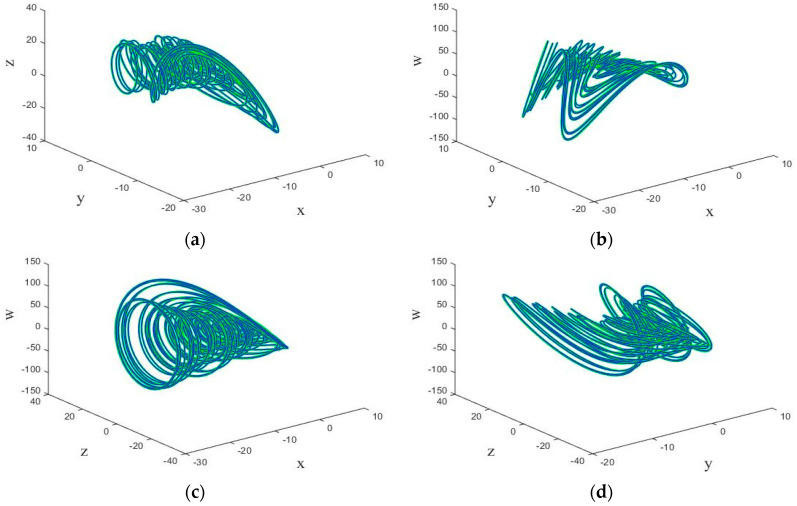
The three-dimensional (3D) chaotic portrait for system (1) in (**a**) *x*-*y*-*z* space, (**b**) *x*-*y*-*w* space, (**c**) *x*-*z*-*w* space, and (**d**) *y*-*z*-*w* space.

The system (1) exhibits periodic and chaotic behavior for different value of g. When g=1.15, the Lyapunov dimension can be calculated by the Kaplan-Yorke dimension. In this case, by taking a=1.05, b=0.7, c=0.19, e=1.37, and f=1.79, the Lyapunov exponent are as $L_{1}=0.185$, $L_{2}=0$, $L_{3}=-0.195$, and $L_{4}=-1.034$. So, the system shows a chaotic behavior. The phase portrait of the chaotic behavior of the system (1) is shown by Figure 2.

**Figure 3:**
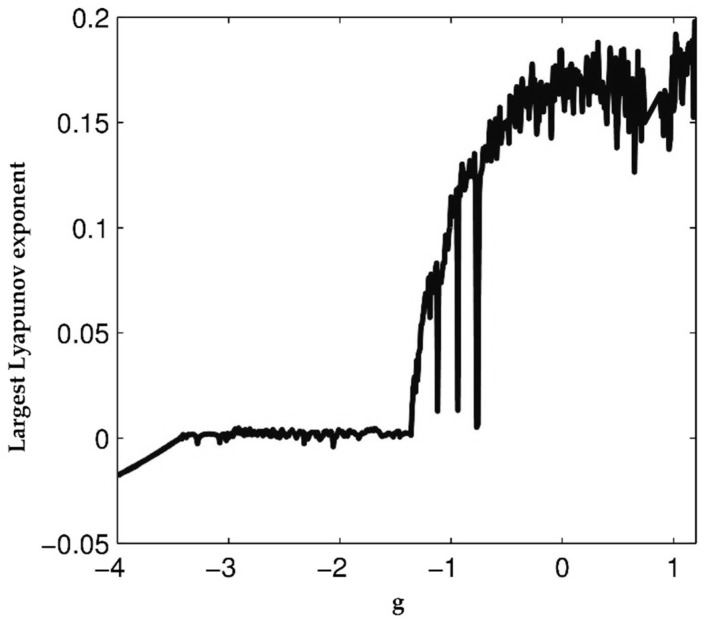
The largest Lyapunov exponent of the system (1).

The largest Lyapunov exponent of the system (1) for −4<g<1.2, a=1.05, b=0.7, c=0.19, e=1.37, and f=1.79 is shown by Figure 3.

Now, for a better understanding of the dynamic characteristics of system (1), its entropy has been analyzed by numerical simulation.

## 3. Entropy Analysis

As well as the positive largest Lyapunov exponent, entropy has been widely used to characterize chaotic systems [53]. This measure focuses on estimating seemingly unpredictable time evolution of chaotic systems and consequently tries to flesh out chaos in terms of randomness [54]. Thus, higher entropy indicates less predictability and a closer approach to stochastic behavior [55]. Although this information can be obtained through theoretical measures, such as Kolmogorov-Sinai entropy, they are often difficult to estimate from a finite data set [56]. Hence, some practical measures to estimate entropy of an underlying system from observed data have been developed in the last years, such as the well-established approximate entropy (ApEn) [57]. This metric has been widely used to characterize dynamical systems [58,59] because it is able to deal with short and noise data with outliers [60]. Briefly, ApEn quantifies times series regularity by computing repetitiveness of similar patterns and provides larger positive values for more irregular data. Hence, considering a N sample-length time series x(n)={x(1),x(1),…,x(N)}, this metric computation requires the following steps:

1. Form N−m+1 *m*-sample length vectors, $X_{m}\left(1\right),\ldots ,X_{m}\left(N-m+1\right)$, defined by $X_{m}\left(i\right)=\left\{x\left(i\right),x\left(i+1\right),\ldots ,x\left(i+m-1\right)\right\}$, for 1≤i≤N−m+1. Each vector contains m consecutive points from the *i*th sample.
2. Compute the Chebyshev distance for any pair of vectors $X_{m}\left(i\right)$ and $X_{m}\left(j\right)$. This distance is defined as the maximum absolute magnitude of the differences between coordinates, i.e.,
  (2) $$d_{ij}^{m}=\underset{k=0,\ldots ,m-1}{\max}\left(\left|x\left(i+k\right)-x\left(j+k\right)\right|\right)$$
3. Estimate the number of pairs of vectors, $X_{m}\left(j\right)$, whose distance with $X_{m}\left(i\right)$ is less than or equal to *r*, i.e.,
  (3) $$C_{i}^{m}\left(r\right)=\frac{1}{N-m+1}\sum_{j=1}^{N-m+1}\Theta \left(r-d_{ij}^{m}\right)$$
  Θ(z) being the Heaviside function, i.e., Θ(z)=1 for z≥0 and Θ(z)=1 for z<0.
4. Calculate the global probability that any two sequences of size *m* present a distance lower than *r*, i.e.,
  (4) $$\emptyset^{m}\left(r\right)=\frac{1}{N-m+1}\sum_{i=1}^{N-m+1}\ln C_{i}^{m}\left(r\right)$$
5. Recompute the steps 1–4 for vectors with *m*+1 samples in length. In this case, Equations (3) and (4) should be replaced by
  (5) $$C_{i}^{m+1}\left(r\right)=\frac{1}{N-m}\sum_{j=1}^{N-m}\Theta \left(r-d_{ij}^{m+1}\right)\;\mathrm{and}\;\emptyset^{m+1}\left(r\right)=\frac{1}{N-m}\sum_{i=1}^{N-m}\ln C_{i}^{m+1}\left(r\right),$$
  respectively.
6. Finally, ApEn can be computed by the difference
  (6) $$\mathrm{ApEn}\left(m,r,n\right)=\emptyset^{m}\left(r\right)-\emptyset^{m+1}\left(r\right)$$

It is well known that this metric presents two limitations, such as it lacks relative consistency and is strongly dependent on the data length [61]. Indeed, when short times series are analyzed ApEn often provides lower values than expected [62]. These limitations have been overcome in its modified version proposed by Richman & Moorman and named sample entropy (SampEn) [61]. This new index presents two main differences from ApEn, i.e.,: (i) self-matches are excluded and (ii) a template-wise strategy is not used. Consequently, N−m vectors of size *m* and *m*+1, for 1≤i≤N−m, are analyzed to compute SampEn, such that new Equations (3)–(5) can be expressed as

(7) $$C_{i}^{m}\left(r\right)=\frac{1}{N-m-1}\sum_{j=1,j\neq i}^{N-m}\Theta \left(r-d_{ij}^{m}\right),\;\emptyset^{m}\left(r\right)=\frac{1}{N-m}\sum_{i=1}^{N-m}C_{i}^{m}\left(r\right),$$

(8) $$C_{i}^{m+1}\left(r\right)=\frac{1}{N-m-1}\sum_{j=1,j\neq i}^{N-m}\Theta \left(r-d_{ij}^{m+1}\right),\;\mathrm{and}\;\emptyset^{m+1}\left(r\right)=\frac{1}{N-m}\sum_{i=1}^{N-m}C_{i}^{m+1}\left(r\right),$$

respectively. As a final step, SampEn can be estimated as

(9) $$\mathrm{SampEn}\left(m,r,N\right)=-\ln\left[\frac{\emptyset^{m+1}\left(r\right)}{\emptyset^{m}\left(r\right)}\right].$$

Chen et al. [63] have proposed a modification of SampEn to avoid a poor statistical stability in some cases due to the binary classification of vectors achieved by the Heaviside function. This new index, named Fuzzy entropy (FuzzEn), considers a smoother definition of a vector match by using a family of exponential functions $D_{ij}^{m}\left(r,k\right)=\exp\left(-{\left(d_{ij}^{m}/r\right)}^{k}\right)$. To quantify the similarity degree among patterns. Thus, Equations (7) and (8) are redefined as

(10) $$C_{i}^{m}\left(r,k\right)=\frac{1}{N-m-1}\sum_{j=1,j\neq i}^{N-m}D_{ij}^{m}\left(r,k\right),\;\mathrm{and}\;C_{i}^{m+1}\left(r,k\right)=\frac{1}{N-m-1}\sum_{j=1,j\neq i}^{N-m}D_{ij}^{m+1}\left(r,k\right),\;$$

respectively. Additionally, the mean from each vector $X_{m}\left(i\right)$ is removed to highlight the local features of the data [63], thus resulting in

(11) $$X_{m}^{*}\left(i\right)=\left\{x\left(i\right),x\left(i+1\right),\ldots ,x\left(i+m-1\right)\right\}-\frac{1}{m}\sum_{l=0}^{m-1}x\left(i+1\right)$$

**Figure 4:**
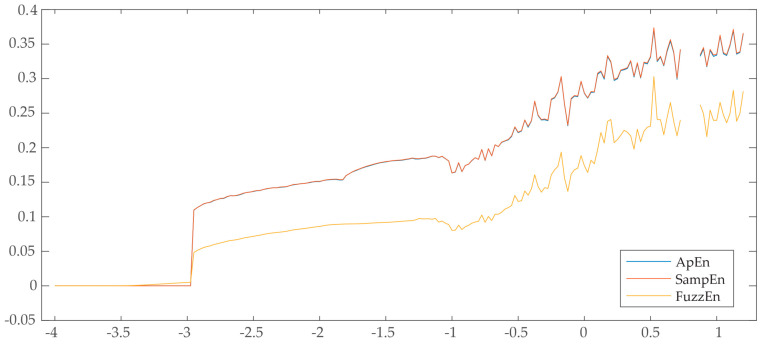
Values of ApEn, SampEn, and FuzzEn computed from x(n) of the system (1) with respect to parameter g.

Clearly, the selection of parameters m and r has a strong impact on the entropy estimates obtained by these three indices. Although no widespread rules exist for their optimal choice, some previous works have recommended the use of m=1 or 2 and *r* between 0.05 and 0.25 times the standard deviation of the data [57,61]. Thus, making use of m=2, r=0.15, and k=2, the values of ApEn, SampEn, and FuzzEn computed from the times series x(n) of the system (1) with length N=3000 are displayed in Figure 4. As can be seen, the three entropy measures provided similar results. In fact, no perceptible differences can be noticed between ApEn and SampEn. Moreover, although FuzzEn revealed lower values than ApEn and SampEn, the same trend can be observed as a function of *g*. To this last respect, entropy shows low values when the system is in a stable state (i.e., for *g* ≤ −1.2) and, contrarily, high values when the system is in a chaotic state (i.e., for *g* > −1.2). The higher the entropy, the higher the degree of uncertainty in the time series, thus requiring more level of information to keep system (1) in a stable state. Note that the large differences between values of ApEn/SampEn and FuzzEn are provoked by their different ways of estimating vector match. Thus, whereas all pairs of vectors presenting a distance larger than *r* do not contribute to entropy computation in ApEn/SampEn [61], FuzzEn always considers the degree of similarity between these patterns, thus obtaining more continuous and smooth entropy estimates [63].

## 4. Brief Review of the RBF-NNs

The objective of control method is to derive the control input for stabilizing the four-dimensional chaotic system (1). Due to their inherent functional approximation and learning capabilities, RBF-NNs have recently received significant attention for approximation and modeling nonlinear functions [46,47]. According to the universal approximation property of the RBF-NN, it can approximate any continuous function $f(x):R^{i}\to R$ with an arbitrary accuracy δ in the following form:

(12) $$f(x)=\theta^{T}\varphi (x)+\delta (x)\;\left|\delta (x)\right|\leq \bar{\delta }$$

where $\theta \in R^{l}$ represents the ideal weight vector, δ(x) denotes the approximation error, and l is the number of neurons. In (12), the ideal parameter vector $\theta \in R^{l}$ satisfies

(13) $$\theta =\arg\;\underset{\hat{\theta }\in R^{l}}{\min}\left\{\underset{x\in \Omega }{\sup}\left|f(x)-{\hat{\theta }}^{T}\varphi (x)\right|\;\right\}$$

where $\hat{\theta }={\left[{\hat{\theta }}_{1}\;{\hat{\theta }}_{2}\;\cdots \;{\hat{\theta }}_{l}\right]}^{T}\in R^{l}$ is the estimate of the ideal weight vector θ, and $\varphi (x)=\left[\begin{array}{cccc} \varphi_{1}(x) & \varphi_{2}(x) & \cdots & \varphi_{l}(x) \end{array}\right]\in R^{l}$ represents the vector of the basis functions.

It is worthwhile to note that the approximation error δ(x) is not known, but it is bounded, i.e., $\left|\delta (x)\right|\leq \bar{\delta }$.

**Figure 5:**
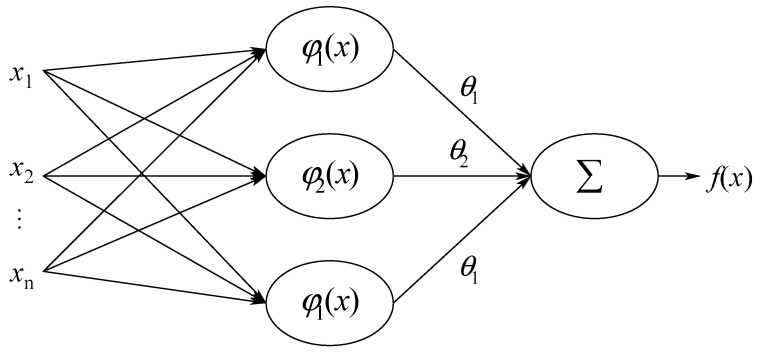
Architecture of the neural network.

In the RBF-NNs, the following well-known Gaussian functions are chosen as the basis functions $\varphi_{j}(x)$ for j=1, 2, …, N

(14) $$\varphi_{j}(x)=e^{-\left(\frac{{\left(x-c_{j}\right)}^{T}\left(x-c_{j}\right)}{\sigma_{j}^{2}}\right)}\;$$

where $c_{j}={\left[\begin{array}{cccc} c_{j,1} & c_{j,2} & \cdots & c_{j,N} \end{array}\right]}^{T}$ and $\sigma_{j}$ denote the center and width of the Gaussian functions, respectively. Figure 5 shows the architecture of the NN.

**Assumption** **1.** *This work assumes that the ideal weight vector has bounded norm, i.e., $\left\|\theta \right\|\leq \bar{\theta }$. However, its bound is unknown.*

**Remark** **1.** *Assumption 1 is only required for the stability analysis and design procedures of the control law does not need $\bar{\theta }$*.

### 4.1. Proposed Adaptive RBF-NN Controller

This section presents the proposed adaptive RBF-NN controller to suppress chaos in the considered system in (1). In the proposed method, all parameters of the system are as unknown as nonlinear dynamics and no prior knowledge about them is available. In order to handle the uncertain nonlinearity, the RBF-NN is invoked to model it. Then, the controller is designed by assuming that the RBF-NN-based model represents the true model of the system. Finally, adaptive learning laws based on the Lyapunov direct method are proposed to tune the adaptive parameters (weights coefficients) of the network.

Before designing the controller, let us rewrite the description of the four-dimensional chaotic system in (1) as follows:

(15) $$\dot{\zeta }=A\zeta +b\left(f(\zeta )+u\right)$$

where $\zeta ={\left[\begin{array}{cccc} \zeta_{1} & \zeta_{2} & \zeta_{3} & \zeta_{4} \end{array}\right]}^{T}\in R^{4\times 1}$ is the state vector, and $\zeta_{1}=x$, $\zeta_{2}=y$, $\zeta_{3}=z$, and $\zeta_{3}=w$; also, $f(\;\zeta )=-a\zeta_{4}+b\zeta_{1}^{2}-c\zeta_{2}^{2}+e\zeta_{1}\zeta_{2}+f\zeta_{1}\zeta_{3}+g$ denotes the uncertain nonlinear dynamics, and $A\in R^{4\times 4}$ and $b\in R^{4\times 1}$ are constant matrices as

(16) $$A=\left[\begin{array}{cccc} 0 & 1 & 0 & 0\\ 0 & 0 & 1 & 0\\ 0 & 0 & 0 & 1\\ 0 & 0 & 0 & 0 \end{array}\right]\;,\;b=\left[\begin{array}{c} 0\\ 0\\ 0\\ 1 \end{array}\right]\;$$

Now, the control input is proposed as

(17) $$u=-\hat{f}(\zeta )+y_{d}^{\left(4\right)}-k^{T}e$$

where $e=\zeta_{1}-y_{d}$ is the tracking error, $e={\left[\begin{array}{cccc} e & \dot{e} & \ddot{e} & \dddot{e} \end{array}\right]}^{T}\in R^{4\times 1}$ represent the error vector and $k={\left[k_{4},\;k_{3},\;k_{2},k_{1}\right]}^{T}\in R^{4\times 1}$ denotes the design parameters that are selected such that all roots of the characteristic polynomial $\Delta (s)=s^{4}+k_{1}s^{3}+k_{2}s^{2}+k_{3}s_{3}+k_{4}$ are in the open left-half of the complex plane.

Now substituting (6) and (7) in (5), we will have

(18) $$\begin{array}{l} \zeta^{\left(4\right)}=f(\zeta )-\hat{f}(\zeta )+y_{d}^{\left(4\right)}-k^{T}e\\ \;={\tilde{\theta }}^{T}\varphi (\zeta )+y_{d}^{\left(4\right)}-k^{T}e+\delta \end{array}$$

where $\tilde{\theta }=\theta -\hat{\theta }$ denotes the parameter approximation error, and adaptive parameters θ are tuned by using the proposed adaptive laws as follows:

(19) $$\dot{\hat{\theta }}=\gamma e^{T}Pb\varphi (\zeta )$$

where γ>0 is the learning rate, and $P\in R^{4\times 4}$ represents a positive definite/semi definite matrix which satisfies the following Riccati-like equation:

(20) $$A_{c}^{T}P+PA_{c}+\sigma P^{T}P+Q=0$$

where $Q\in R^{4\times 4}$ is a positive definite matrix, and σ>0 is a design parameter.

Before presenting stability analysis, the error dynamics is obtained by considering (15) and (18) as

(21) $$\dot{e}=A_{c}e+b{\tilde{\theta }}^{T}\varphi (\zeta +\delta )$$

where

(22) $$A_{c}=\left[\begin{array}{cccc} 0 & 1 & 0 & 0\\ 0 & 0 & 1 & 0\\ 0 & 0 & 0 & 1\\ k_{4} & k_{3} & k_{2} & k_{1} \end{array}\right]$$

Now, stability analysis of the proposed controller is presented by considering the following Lyapunov function:

(23) $$V=\frac{1}{2}e^{T}Pe+\frac{1}{2\gamma }{\tilde{\theta }}^{T}\tilde{\theta }$$

Differentiating (21) with respect to time, results in

(24) $$\dot{V}=\frac{1}{2}{\dot{e}}^{T}Pe+\frac{1}{2}e^{T}P\dot{e}-\frac{1}{\gamma }{\tilde{\theta }}^{T}\dot{\hat{\theta }}$$

Substitution of (19) in (23), results in

(25) $$\begin{array}{l} \dot{V}=\frac{1}{2}\left(e^{T}A_{c}^{T}+\varphi {\left(\zeta \right)}^{T}\tilde{\theta }b^{T}+\delta \right)Pe+\frac{1}{2}e^{T}P\left(A_{c}e+b{\tilde{\theta }}^{T}\varphi (\zeta )+\delta \right)-\frac{1}{\gamma }{\tilde{\theta }}^{T}\dot{\hat{\theta }}\\ \;=\frac{1}{2}e^{T}\left(A_{c}^{T}P+PA_{c}\right)e-\frac{1}{\gamma }{\tilde{\theta }}^{T}\left(\dot{\hat{\theta }}-\gamma e^{T}Pb\varphi (\zeta )\right)+\delta Pe \end{array}$$

Again, substituting the proposed adaptive learning law (19) in (25), yields

(26) $$\begin{array}{l} \dot{V}=\frac{1}{2}e^{T}\left(A_{c}^{T}P+PA_{c}\right)e+\delta Pe\\ \;\leq \frac{1}{2}e^{T}\left(A_{c}^{T}P+PA_{c}+\sigma P^{T}P\right)e+\frac{1}{2\sigma }{\bar{\delta }}^{2}\\ \;\leq -\frac{1}{2}e^{T}Qe+\frac{1}{2\sigma }{\bar{\delta }}^{2}\leq -\frac{1}{2}\underline{\lambda }(Q)e^{T}e+\frac{1}{2\sigma }{\bar{\delta }}^{2} \end{array}$$

where $\underline{\lambda }(Q)$ denotes to the minimum eigenvalue of matrix Q. As it is obtained from (26), the condition $\left\|e\right\|\leq {\bar{\delta }}^{2}/\sigma \underline{\lambda }$ results in $\dot{V}\leq 0$. This inequality shows that all of the closed-loop signals (i.e., e and $\tilde{\theta }$) are semi-globally uniformly ultimately bounded [48].

**Remark** **2.** *The design parameter*σ*in the Riccati-like Equation (20) has been proposed to attenuate the inevitable effects of the approximation error on*$\dot{V}$.

**Remark** **3.** *It should be noted that the proposed controller does not require any off-line learning phase.*

### 4.2. Simulation Results

This section presents some simulation results to investigate the effectiveness of the proposed adaptive RBF-NN-based controller. A typical chaotic behavior of the uncontrolled system was discussed in Section 2. Now, the control objective is to stabilize the considered unknown chaotic system in (1) and to derive it to the equilibrium point.

To design the proposed controller, one RBF-NN composed of 50 neurons was constructed. The center of the membership functions and initial weights of the network were set at 1. For simulation, $\sigma_{i}$ and γ were set to 0.01, and 0.5, respectively, and the initial conditions were chosen as $\zeta (0)={\left[\begin{array}{cccc} 0 & -1 & 0 & -1.5 \end{array}\right]}^{T}$. As mentioned before, the proposed approach does not require any training data and any off-line learning phase. After the construction of the RBF-NN, it is used to model the uncertain function f(ζ) and then the control input (17) is applied. The design parameters $k_{1},\;k_{2},\;k_{3}$ and $k_{4}$ in the control input (17) are chosen such that the all roots of the characteristic polynomial Δ(s) remain in the open left-half of the complex plane. For simulation, these parameters were chosen as $k_{1}=20$, $k_{2}=24$, $k_{3}=25$, and $k_{4}=22$. Also, by solving the Riccati-like Equation (20), the following matrix P was obtained:

(27) $$P=\left[\begin{array}{cccc} 5 & 0 & 0 & 0\\ 0 & 5 & 0 & 0\\ 0 & 0 & 5 & 0\\ 15 & 25 & 20 & 10 \end{array}\right]$$

Also, adjustable parameters $\hat{\theta }\in R^{50}$ was adjusted based on the proposed adaptive learning law in (19).

**Figure 6:**
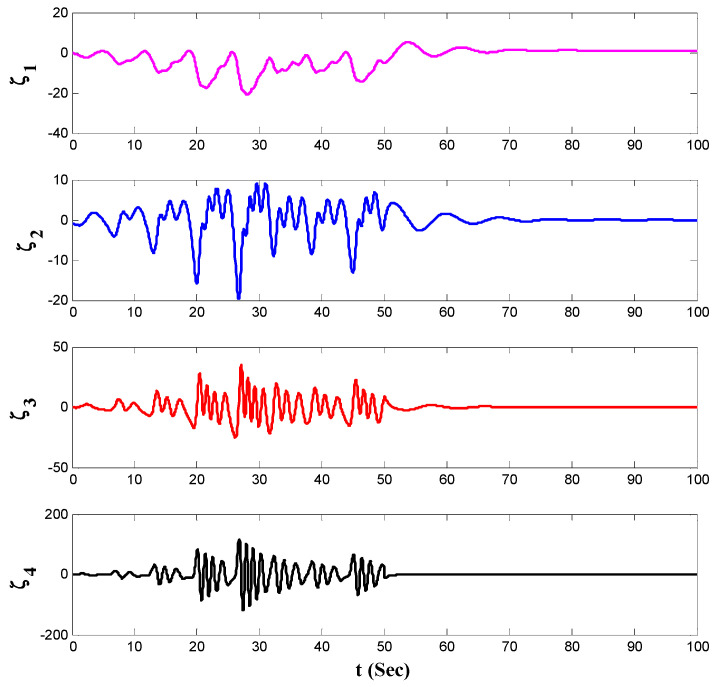
The state variables when the proposed control input is activated at t=50 s.

**Figure 7:**
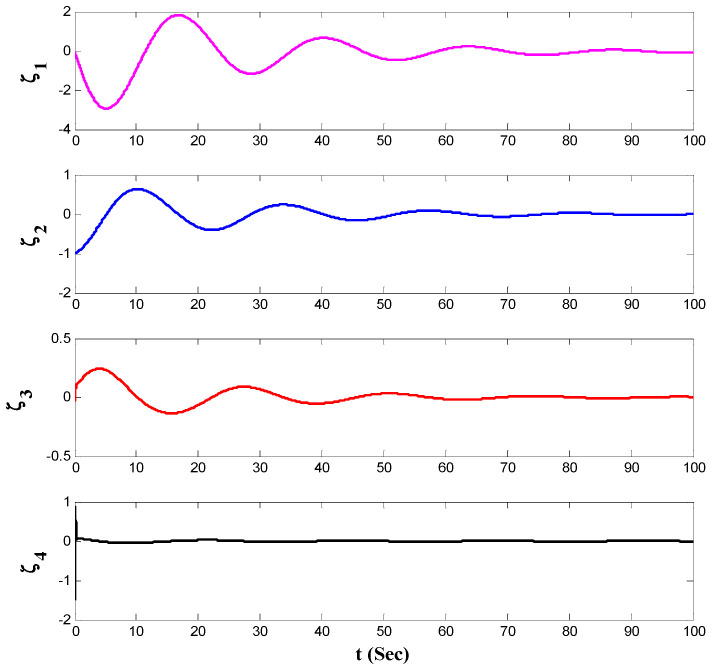
The state variables in the presence of the proposed control method.

**Figure 8:**
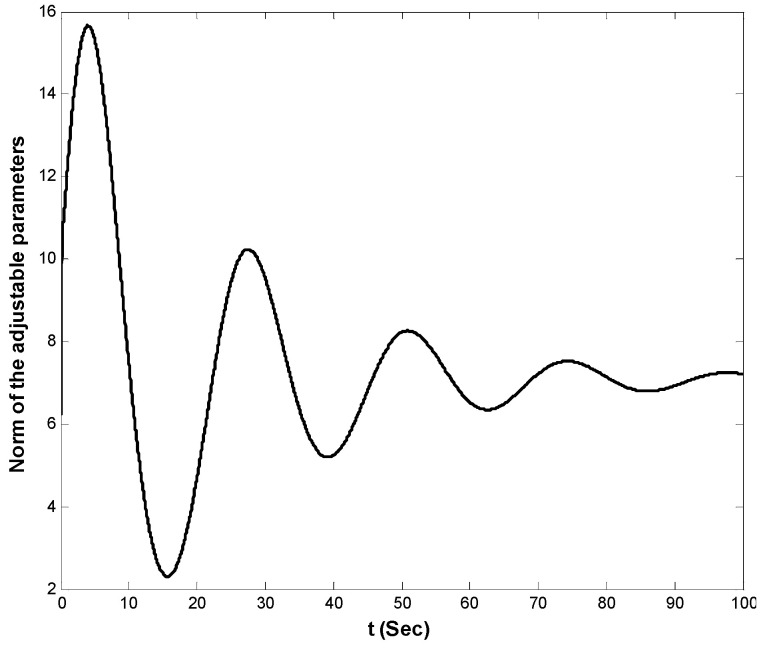
Norm of the weights of the RBF-NN.

**Figure 9:**
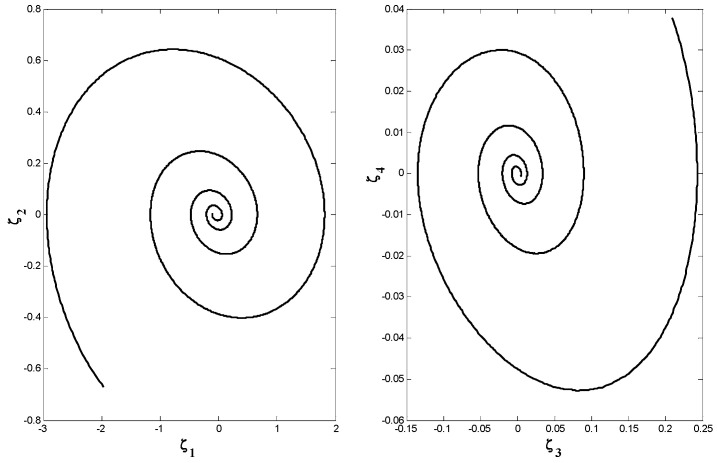
Phase portraits of the controlled system.

**Figure 10:**
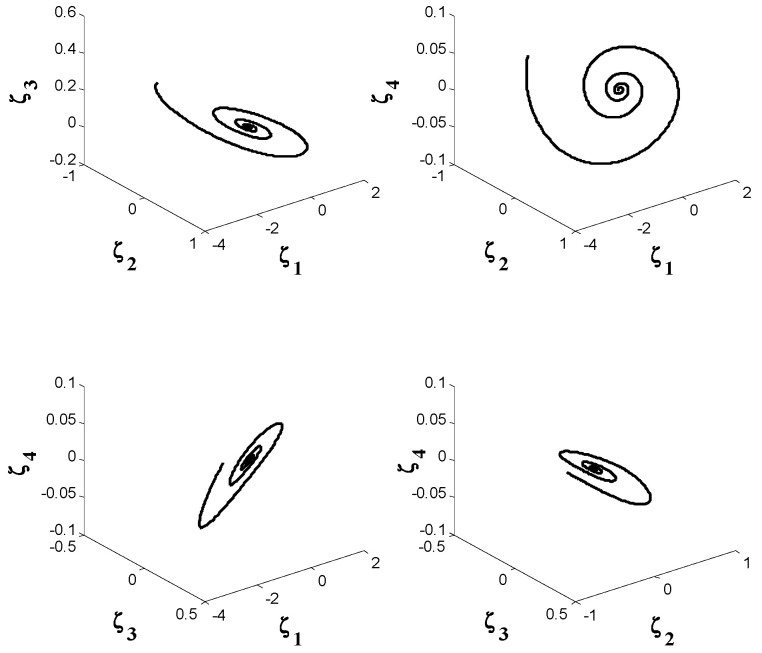
The 3-D behavior of the controlled system.

Figure 6, Figure 7, Figure 8, Figure 9 and Figure 10 depicts the simulation results. To highlight the performance of the proposed approach, at first the control input was set as zero, then after t=50 s the proposed control method was activated. As obtained from the depicted results in Figure 6, before the activation of the proposed controller, the system has chaotic behavior but after the activation of it, the chaos was suppressed, and the desired behavior is obtained.

The state variables of the system by using the proposed controller are shown in Figure 7. Also, norm of the estimated weight coefficients is shown in Figure 8. The obtained result in Figure 8 shows that the norm of the adjustable parameters is bounded. Figure 9 and Figure 10 depict the phase portraits and the three-dimensional behavior of the controlled system, respectively. The reported results demonstrate the ability of the proposed approach to stabilize the considered non-equilibrium four-dimensional chaotic system with hidden attractors.

## 5. Conclusions

In this study, a new adaptive radial basis function-neural network-based control scheme was proposed to stabilize a specific four-dimensional chaotic system, which shows a periodic-double and low-entropy route preceding high-entropy chaotic states. The proposed controller design requires neither any initial information about the dynamics of the chaotic system nor its parameters. The uncertain dynamics of the considered four-dimensional system is approximated by using the RBF-NN, and then the proposed indirect adaptive control law is proposed based on the developed model. Stability analysis is presented, and adaptive learning law is derived for calibrating weights of the RBF-NN. Simulation results verify the acceptable performance of the proposed method for stabilizing the considered chaotic system.
